# Influence of Agropastoral System Components on Mountain Grassland Vulnerability Estimated by Connectivity Loss

**DOI:** 10.1371/journal.pone.0155193

**Published:** 2016-05-12

**Authors:** Maite Gartzia, Federico Fillat, Fernando Pérez-Cabello, Concepción L. Alados

**Affiliations:** 1 Instituto Pirenaico de Ecología (CSIC), Jaca, Huesca, Spain; 2 Department of Geography and Spatial Management, and Aragon University Research Institute in Environmental Science (IUCA) (University of Zaragoza), Zaragoza, Spain; 3 Instituto Pirenaico de Ecología (CSIC), Zaragoza, Spain; Universidade de Aveiro, PORTUGAL

## Abstract

Over the last decades, global changes have altered the structure and properties of natural and semi-natural mountain grasslands. Those changes have contributed to grassland loss mainly through colonization by woody species at low elevations, and increases in biomass and greenness at high elevations. Nevertheless, the interactions between agropastoral components; i.e., ecological (grassland, environmental, and geolocation properties), social, and economic components, and their effects on the grasslands are still poorly understood. We estimated the vulnerability of dense grasslands in the Central Pyrenees, Spain, based on the connectivity loss (CL) among grassland patches that has occurred between the 1980s and the 2000s, as a result of i) an increase in biomass and greenness (CL-IBG), ii) woody encroachment (CL-WE), or iii) a decrease in biomass and greenness (CL-DBG). The environmental and grassland components of the agropastoral system were associated with the three processes, especially CL-IBG and CL-WE, in relation with the succession of vegetation toward climax communities, fostered by land abandonment and exacerbated by climate warming. CL-IBG occurred in pasture units that had a high proportion of dense grasslands and low current livestock pressure. CL-WE was most strongly associated with pasture units that had a high proportion of woody habitat and a large reduction in sheep and goat pressure between the 1930s and the 2000s. The economic component was correlated with the CL-WE and the CL-DBG; specifically, expensive pastures were the most productive and could maintain the highest rates of livestock grazing, which slowed down woody encroachment, but caused grassland degradation and DBG. In addition, CL-DBG was associated with geolocation of grasslands, mainly because livestock tend to graze closer to passable roads and buildings, where they cause grassland degradation. To properly manage the grasslands, an integrated management plan must be developed that includes an understanding of all components of the agropastoral system and takes into account all changes that have occurred in dense mountain grasslands. Addressing the problems individually risks the improvement of some grasslands and the deterioration of others.

## Introduction

Semi-natural mountain grasslands are protected by the EU Habitats Directive and require management, especially through grazing, for their maintenance [1]. The environmental factors and management practices that allowed the creation of those grasslands have changed [2, 3]. If those habitats are misused or mismanaged, they will disappear, either through succession toward the climax vegetation [4–6] or through degradation [7]. Global changes affect grassland resilience (“the capacity of a system to absorb disturbance and reorganize while undergoing change so as to still retain essentially the same function, structure, identity, and feedbacks” [8]), and increase their vulnerability to environmental pressures [9, 10]. Once a resilience threshold has been exceeded, the grassland structure changes [11–13], through increases in woody encroachment, changes in biomass and greenness [8, 14, 15], or connectivity loss among vegetation communities [5, 16]. Persistence of traditional land uses is therefore vital for the preservation of those grasslands [17].

The ecosystem services that mountain grasslands provide are important to human wellbeing [18, 19], and these grasslands are being lost rapidly [20–23]. Less well understood are the relationships between the components of agropastoral systems (economic, social, and ecological) and the specific changes that have occurred in mountain grassland ecosystems, which are important for identifying the proper management of these habitats [24]. The interactions between the components of an agropastoral system define the type of agropastoral activities that can occur in an area [25], and these components dictate the state of mountain grasslands. For example, environmental factors like topography and climate of a region determine the quality and productivity of the grassland, the species composition of livestock (e.g., sheep, goats, cattle, equids), and the number of livestock that can be sustained efficiently [5, 26–28]. From an economic perspective other factors are important; e.g., livestock productivity, market price, availability of stockbreeders and their dependence on off-farm jobs for income [5]. In addition, even under similar economic and ecological conditions, differences in social circumstances can influence the course of events. For example, in highly populated rural areas with high availability of labor, grassland and livestock management will differ from that in sparsely populated rural areas, where agricultural pressures are low. To fully understand an agropastoral system and the proper way to manage it, an understanding of present and past socioeconomic and ecological components is necessary [29–31].

Early in the 20th century most of the inhabitants of rural areas in the Spanish Pyrenees were stockbreeders [11], who shepherded small flocks of sheep and goats [32]. At that time, livelihoods in rural areas were based on the exploitation of natural resources. In the mid-20th century many inhabitants abandoned rural areas and moved to industrialized cities, and only a few stockbreeders that had few livestock remained to use and manage the grasslands [33]. By the end of the 20th century the stockbreeders remaining in the area had more livestock units, most of which were cattle, than they would have had in the past [15, 32]. In rural areas that had been abandoned decades earlier, income from tourism has increased (e.g., national parks and ski resorts) and most of those stockbreeders have nowadays an off-farm job in the service sector [11]. Thus, stockbreeder activity has decreased [5, 17, 34], changing the economic and social structure of rural areas [11, 32, 33].

The principal objective of this study is to identify the components of the agropastoral system associated with the changes that occurred between the 1980s and the 2000s in dense mountain grasslands of the Central Pyrenees, Spain. The specific objectives are (i) to measure the connectivity loss of grasslands caused by 1) increases in biomass and greenness (CL-IBG), 2) woody encroachment (CL-WE), and 3) decreases in biomass and greenness (CL-DBG), and (ii) to quantify the relationships between connectivity loss and the economic, social, and ecological (environmental, grassland, and geolocation) components of the agropastoral system. Identifying the mechanisms underlying change in grassland connectivity loss will inform management practices to counteract those changes.

We hypothesized that all socioeconomic and ecological components would affect grasslands, although the direction and intensity of these effects may differ considerably. Given the current socioeconomic circumstances, characterized by the abandonment of some areas and the overuse of others [2], the environmental and grassland components may have the greatest effect [3, 28, 35]. The environmental component will dominate in areas where abandonment has occurred [36], as it is defined by factors that do not change over time (e.g., lithology, topography) that dictate the seriation toward climax vegetation. After land abandonment, the grassland component associated to livestock pressure could retard seriation. Conversely, some areas were expected to become intensified, leading to grassland degradation; in these cases, the geolocation component (e.g., distance to nearest building or road) could be the primary driver involved. Changes in the economic component, mainly through changes in the dedication of stockbreeders to agropastoral activities because of increases of part-time jobs, typically in the tourism sector, could drive decreases in agropastoral activities and influence land use pressure and the extent of land abandonment [17, 37]. Regarding the social component, we expect it to drive changes in grasslands through changes in grassland management.

## Methods

### Ethics statement

Some of the data were obtained from semi-structured, in-person interviews with the 201 stockbreeders who used the summer pasture units (SPU), conducted between 2010 and 2013. Written consent was not requested because interviews were anonymous and voluntary, and the data were analyzed collectively, not individually, which maintained participant confidentiality. Stockbreeder responses were recorded on paper, not by audio recording. Only six of 201 interviewees refused to proceed with the interview; all others gave verbal consent to participate in the study. The academic board of the doctoral program in Spatial Planning and Environment at the Universidad de Zaragoza (Spain) approved the study.

No specific permission was required from the communities included in the study; all data were collected from the interviews or public databases (via websites). We confirm that field studies did not involve endangered or protected species. In addition, the Instituto Pirenaico de Ecología (Consejo Superior de Investigaciones Científicas in Spain) approved all procedures.

### Study area

**Fig 1:**
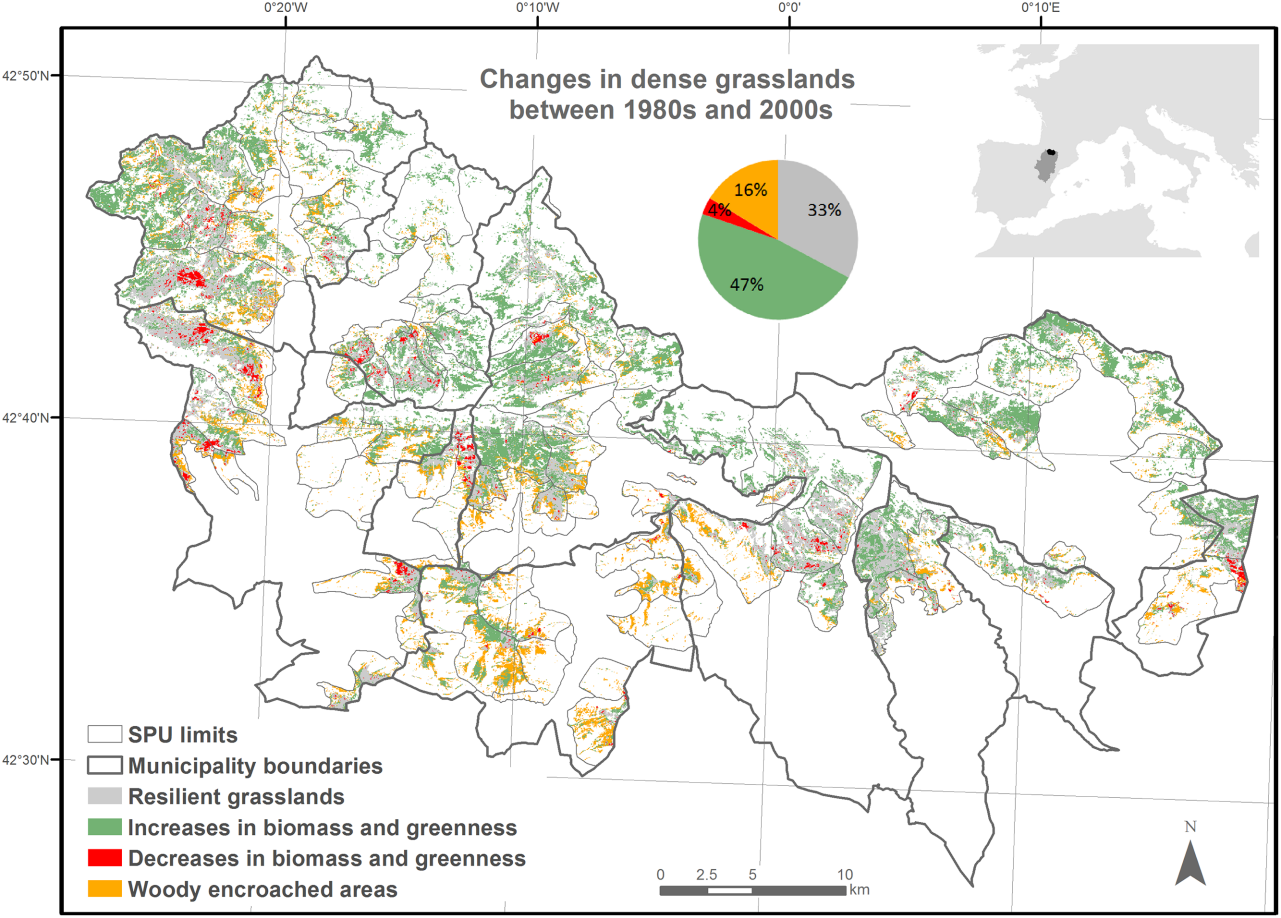
Study area in the Central Pyrenees, Aragón, Spain. The study was restricted to the dense grasslands that were present in the 1980s within the summer pasture units (SPU). The percentage indicates the proportions of the grasslands that have changed or have remained unchanged.

The study area encompassed 11 municipalities in the Central Pyrenees, Aragón, Spain, in Alto Gállego and Sobrarbe Counties (42° 36´ N, 0° 00´E) (Fig 1). At least since the Middle Ages, the area has been used for traditional grazing activities [38]. In the 1930s, the population of rural areas in the Pyrenees began to decline and, consequently, so did grazing activities [15]. Before that, mountain grasslands were grazed mainly by small (around 300 heads) flocks of sheep and goats, which were managed by shepherds. The sheep were *Churra Tensina*, a highly rustic, adaptable, versatile, and productive breed that provided meat, milk, wool, and leather. By the 1980s, the number of sheep and goat heads in the area had decreased by 80%, and numbers have continued to decline [15]. In addition, *Rasa Aragonesa* became the most popular sheep breed. Since 1960, the number of cattle has increased by 243%, as farms went from having a few cows (2–3 heads) of the rustic *Pirenaica* breed to large herds of *Parda* cattle. Even so, since 1930s, the number of large livestock units declined from 15,000 to 9,000 in the municipalities of the study area [15, 33]. Thus, changes in the grasslands in recent decades likely have their origin in the socioeconomic changes that began in the 1930s.

The study area has a mountain climate that differs considerably depending on elevation (600 m—3355 m), aspect (north-south gradient), and the presence of Cantabric and Continental-Mediterranean influences (west-east gradient). Mean annual precipitation ranges from 866 to 1772 mm, and mean minimum temperature ranges from 5.8°C in the lowlands to 0.7°C in the highlands [39]. In recent decades, mean maximum temperatures have increased, and precipitation and snow accumulation have decreased in the Pyrenees [15, 40–42], and further increases in temperature have been predicted in the region [43]. The lithology of the study area varies considerably. Most (68%) of the area lies on acidic materials (sand and clay, slate and quartzite, or granite), 26% on basic materials (limestone or marl), and 6% on quaternary materials (alluvium, colluvium, or moraines), which occur at the bottom of the valleys (see S6 Fig for the SPU areas)[44].

Climatic variation along elevation and lithology influence the types of vegetation in the study area. Nineteen percent of the study area is dominated by sparse grasslands (<50% vegetation cover, on steep slopes, shallow soil, rocky outcrops, mostly in the alpine region) and 22% by dense grasslands (>50% vegetation cover, in flatter areas, deeper soil, mostly in the subalpine region). Forests (32%) and shrubs (25%) predominated below the subalpine region [14].

The study included 92 summer pasture units (SPU); i.e., the traditional land-use partitioning of summer grazing pastures among the mountain grasslands of the study area. Within the SPUs we focused on dense grasslands, which covered 26,950 ha. These natural and semi-natural mountain grasslands are under European Union protection, as described in Annex I of Council Directive 92/43/EEC for the conservation of natural habitats and of wild fauna and flora [45–47]. Specifically, the communities studied here include siliceous Pyrenean *Festuca eskia* grasslands (6140), semi-natural dry grasslands and scrubland facies on calcareous substrates (*Festuco-Brometalia*, with important orchid sites of conservation priority, 6210), and the species-rich *Nardus* grasslands (on siliceous substrates in mountain areas and submountain areas in Continental Europe, 6230). The seriation of the grasslands toward climax vegetation as a response to land abandonment (e.g., low grazing pressure from domestic herbivores) and climate warming is expected to increase the biomass and greenness of the natural and semi-natural grasslands. In the semi-natural grasslands, seriation proceeds toward woody habitats, and this process happens faster close to woody habitats [14].

In the study area, 16% of the dense grasslands experienced woody encroachment between the 1980s and the 2000s [14]. In this period, grassland biomass and greenness increased in extent by 47% and decreased 4% respectively [15]. A third of the grasslands were classified as resilient because they did not change significantly between the 1980s and the 2000s [14, 15](Fig 1).

### Measurements of physiognomic and physiologic properties of grasslands

The amounts of **woody-encroached** (WE) areas within dense grasslands were estimated based on Landsat-5 TM imagery taken in the 1980s and the 2000s [14], which were provided by the USGS at level 1T (ortho-rectified image) and correspond to scene 199–30 [48]. After radiometric correction, the images from early and late summer were combined with auxiliary data and used in the supervised classification to identify the land cover types in the study area: forest, shrubland, dense and sparse grasslands, and cultivated areas [14, 49]. The transition probability matrix based on the maps from the two periods was used to quantify the extent of changes that occurred from dense grasslands in the 1980s towards woody habitat in the 2000s [14].

To quantify the changes in **biomass and greenness** (increases and decreases in biomass and greenness: IBG and DBG) in the dense grasslands that persisted from the 1980s to the 2000s, we measured the Normalized Difference Vegetation Index (NDVI) [50] and Normalized Difference Infrared Index (NDII) [51] based on the Landsat-5 TM imagery taken in the 1980s and the 2000s [15]. These indices are positively correlated with vegetation biomass and greenness, respectively [52, 53], and were combined to create multitemporal vectors, which were used to detect changes [15, 54]. The intensity of changes was calculated based on the absolute differences in the two indices between the two periods; changes could be positive or negative depending on the direction of the changes of the indices. If both indices increased, there had been an IBG, and if they decreased, there had been a DBG [15].

**Fig 2:**
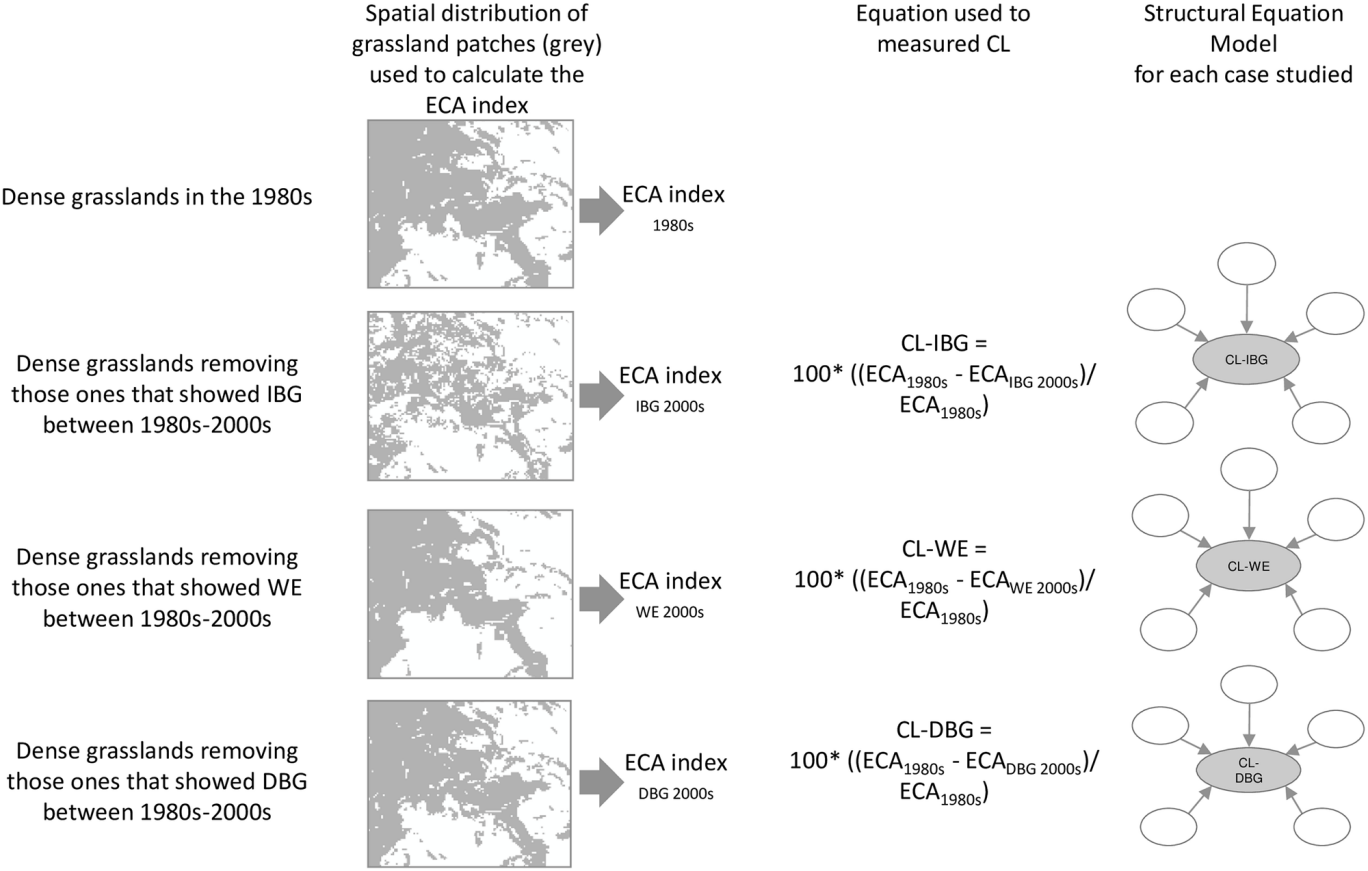
Diagram of the methodology used to measure connectivity loss (CL) in dense mountain grasslands. The spatial distribution of the patches represents a small portion of the study area. (IBG = increase in biomass and greenness, WE = woody encroachment, DBG = decrease in biomass and greenness, ECA = Equivalent Connected Area).

To measure connectivity loss (CL) of dense grasslands caused by IBG, WE, and DBG, we calculated the Equivalent Connected Area (ECA) index [55] for the 1980s and the 2000s (Fig 2). This index is based on the probability of connectivity, which takes into account habitat availability and spatial graph structures, and intra- and inter-patch connectivity at the landscape scale [55, 56]. The structural connectivity of grasslands was measured without taking into account any impediments for species movement. For the 1980s, the ECA index was based on all dense grassland patches (Fig 2); for the 2000s, one ECA index was calculated for each IBG, WE, and DBG (Fig 2). In each case, we removed grassland patches that had undergone IBG, WE, or DBG, and the ECA indices were calculated based on the remaining grassland patches. The difference in the index between the two periods (dECA) for each SPU reflected the change in connectivity (Fig 2). In the calculation of the probability of connectivity, we used 30 m as the dispersal distance because a pixel in the images represented a 30 m x 30 m area. Therefore, when a pixel changes between periods, we detect connectivity loss in grassland units. ECA indices were calculated using Conefor 2.6 software [57, 58].

### Statistical analyses

**Fig 3:**
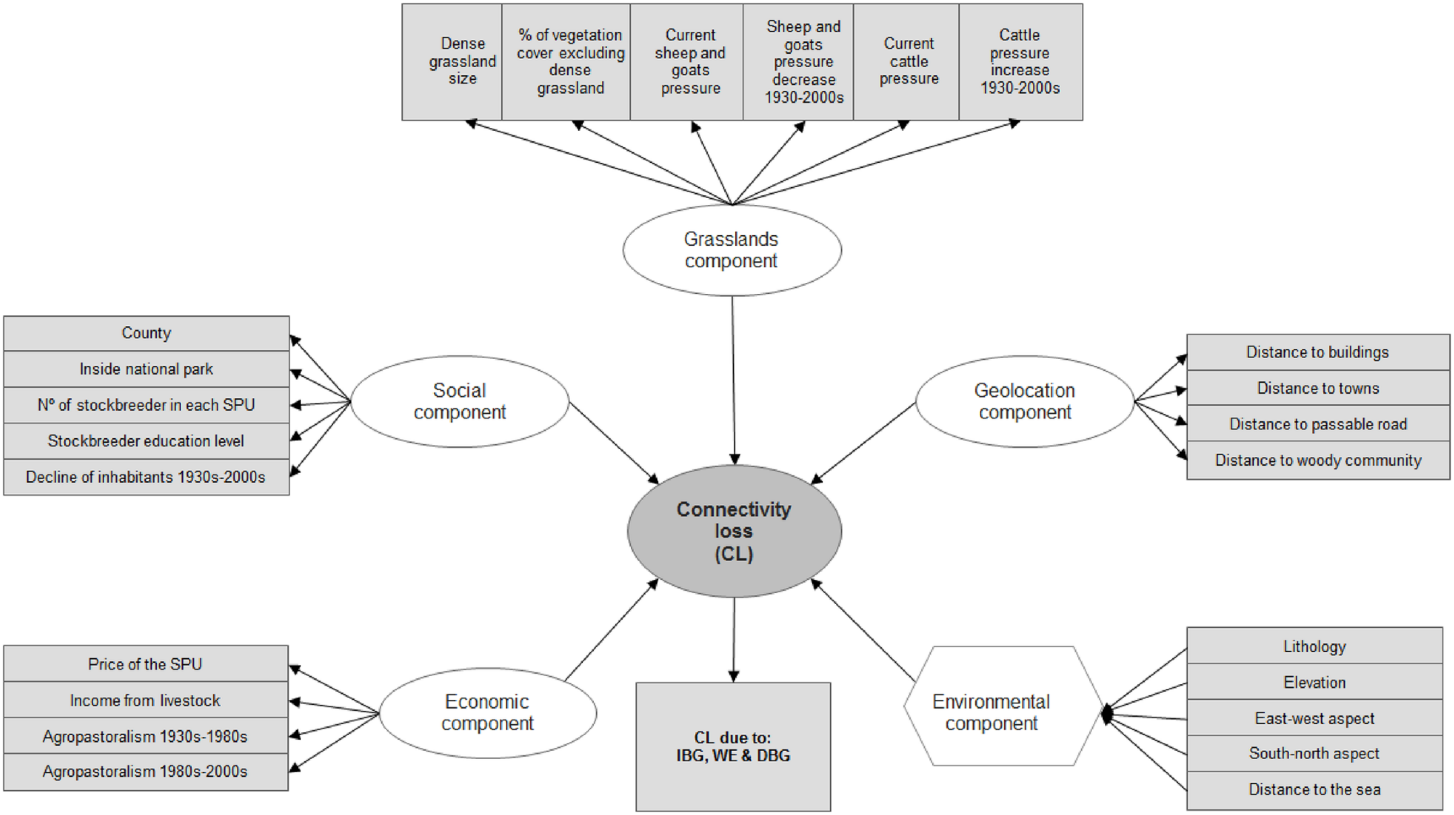
Conceptual structural equation model of the theoretical agropastoral system framework. The components of the agropastoral system can affect the connectivity loss in dense grasslands. Latent variables are indicated by white ellipses and composite variables by hexagons; rectangles indicate the manifest (observed) variables with which the latent and composite variables were defined.

To quantify the relationships between the agropastoral system components (economic, social, grassland, geolocation, and environmental) and the connectivity loss (CL) in the dense grasslands at the SPU level, we used Structural Equation Modeling (SEM) [59] (Fig 3). SEM models were generated for CL-IBG, CL-WE, and CL-DBG. SEM can use manifest (observed and directly measured) variables to understand latent (unobserved) variables and build composite variables [60, 61] (Fig 3). In our study the agropastoral system components were included as latent variables; these variables “represent a factor we believe to exist and to be relevant to our analysis, but which are not measured directly” and are described by different manifest variables in the same dimension [61]. An exception was the environmental component, which was a composite variable based on observed variables that described each SPU and represented potentially heterogeneous collections of causal factors [61] (Fig 3).

SEM was performed using the PLSPM (Partial Least Squares Path Modeling) statistical approach, which is useful for modeling complex multivariable relationships [62]. To assess the overall model, we used the Goodness of Fit (GoF) index. The structural model was evaluated based on the R^2^ fit index. To confirm whether the models’ assumptions were met, we used the Dillon-Goldstein’s Rho index (D.G. Rho) to test the unidimensionality of the manifest variables in each of the latent variables. Only the variables that had a D.G. Rho > 0.7 were used. For each of the latent variables we included only those manifest variables that had low collinearity (Pearson r < 0.7). All statistical analyses were performed using XLSTAT software.

### Agropastoral system components defined by manifest variables

At the SPU level, we assessed the relationships between the economic, social, grassland, geolocation, and environmental components of the agropastoral system and the connectivity loss in the dense grasslands (Fig 3). Each component represented as a latent variable was the result of several manifest variables that were measured in the study area (Fig 3). The results are presented as mean ± SE.

The **grassland component** was based on the following manifest variables (S1 and S2 Figs):

- Dense grassland size: hectares of dense grassland in each SPU, each assigned to one of three categories: SPU > 300 ha (value in the model = 1), 300–100 ha (value in the model = 2), and < 100 ha (value in the model = 3).
- Proportion (%) of the vegetation cover that did not include dense grassland communities, which was measured as 100* (1- (dense grassland ha in each SPU / total ha of each SPU)).
- Current sheep and goat, and cattle pressures in each SPU (data from stockbreeder interviews) were expressed as large livestock units (LLU) multiplied by the number of months that the livestock spent in each SPU per hectare of grassland. To convert the number of livestock to LLU, we multiplied the number of sheep and goat heads by 0.125, and the number of cattle by 0.8 [63]. We assumed that sheep and goats grazed both the dense and the sparse grasslands, and cattle used the dense grasslands only [64]. In the calculations of livestock pressure, we took into account that each livestock type uses different grasslands.
- Percentage of change in the number of livestock heads between the 1930s and the 2000s at the municipality level [65]: decrease (%) in sheep and goats = 100* ((number of sheep and goat heads in the 1930s—number of sheep and goat heads in 2000s) / number of sheep and goat heads in the 1930s); increase (%) in cattle = 100* ((number of cattle heads in 2000s—number of cattle heads in the 1930s) / number of cattle heads in the 1930s)

The **social component** was based on the following manifest variables (S3 Fig):

- County: Alto Gállego, which had ski resort-related tourism (value in the model = 0), and Sobrarbe, which had national park-related tourism (value in the model = 1).
- SPU inside (value in the model = 1) or outside (value in the model = 0) the national park.
- Number of stockbreeders using each SPU (data from interviews)
- Stockbreeder education level in each SPU (data from interviews): no formal education (value in the model = 1), primary education (value in the model = 2), secondary or higher education (value in the model = 3), and cases in which an SPU was not used by any stockbreeder (value in the model = no data)
- Decrease (%) in the number of inhabitants at the municipality level between the 1930s and the 2000s [65], which was calculated as follows: 100* ((number of inhabitants in the 1930s—number of inhabitants in the 2000s) / number of inhabitants in the 1930s)

The **economic component** was based on the following manifest variables (S4 Fig):

- The stockbreeder’s opinion of the price that he or she paid to use the SPU (data from interviews): when no stockbreeders used the SPU (value in the model = 0), reasonable price (value in the model = 1), reasonable-high price (value in the model = 2), or high price (value in the model = 3)
- Income from livestock (data from interviews): proportion (%) of the stockbreeder’s income that was derived from livestock breeding: 0% (value in the model = 0), 1%—25% (value in the model = 1), 26–50% (value in the model = 2), 51–75% (value in the model = 3), 76–100% (value in the model = 4)
- Importance of the agropastoral sector in each municipality between the 1900s and the 1980s, and between the 1980s and the 2000s (value range = -100 to 0; -100 = no agropastoral sector [65]). The importance of the agropastoral sector was estimated based on the amount of employment in the primary sector relative to the employment in all other sectors, as follows: agropastoralism in the period 1930s—1980s = 100* (primary sector employees in the 1980s—primary sector employees in the 1930s) / primary sector employees in the 1930s)); agropastoralism in the period 1980s—2000s = 100* (primary sector employees in the 2000s—primary sector employees in the 1980s) / primary sector employees in the 1980s))

The **geolocation component** was based on the following manifest variables (S5 Fig): average distance from the SPU to the nearest building, town, passable road, and woody habitat [44, 66].

The **environmental component** was based on the following manifest variables (S6 Fig): a composite variable that included lithology (% of basic and quaternary material), topography (the median elevation, east-west aspect (sine of the aspect), south-north aspect (inverse of the cosine of the aspect)) and climate (median distance to the sea from each SPU, which reflected the degree of continentality) [44].

## Results

### Connectivity loss in the dense grasslands

**Fig 4:**
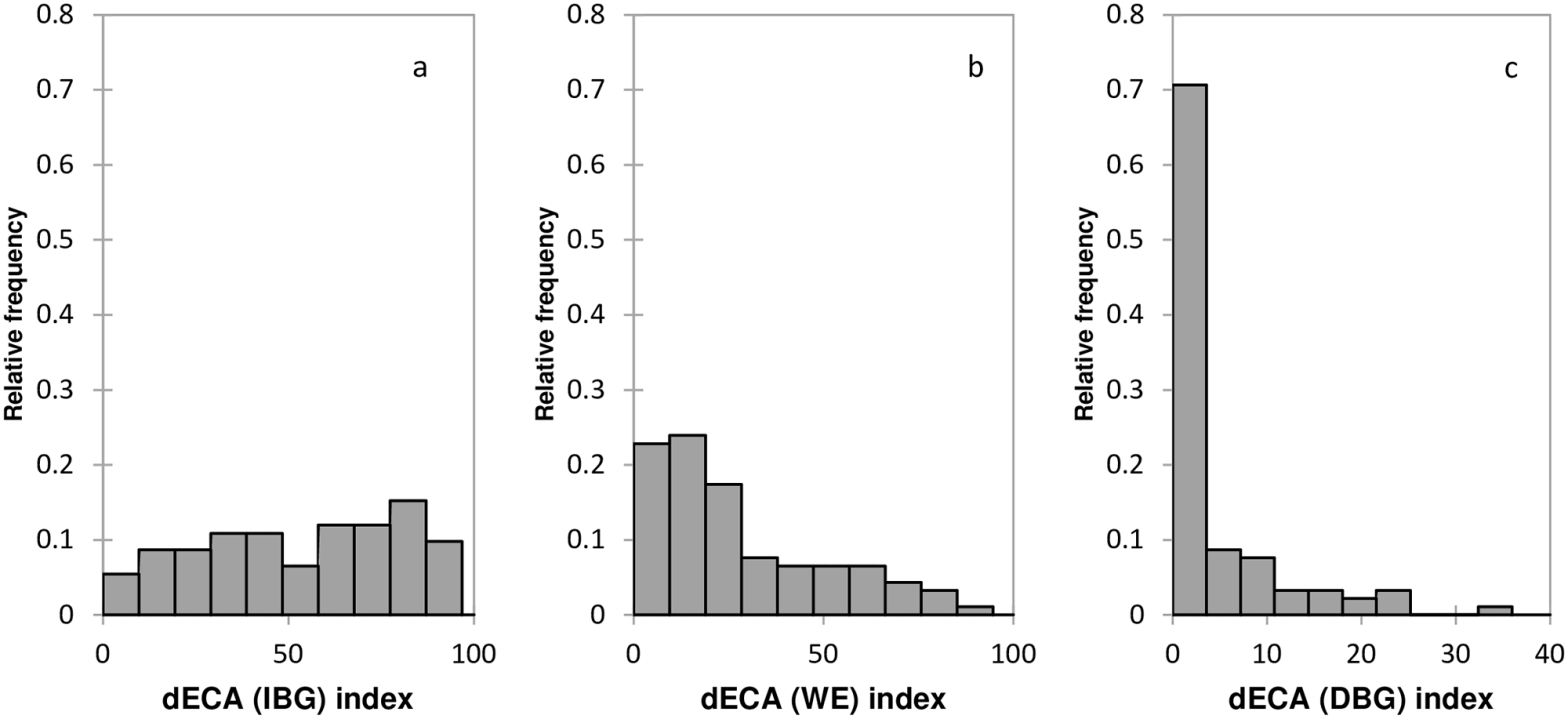
The relative frequencies of summer pasture units (SPU) that lost grassland connectivity caused by a) IBG, b) WE, and c) DGB, as estimated by the dECA index.

In the dense mountain grasslands of the Central Pyrenees, Spain, between the 1980s and the 2000s, all of the 92 SPU experienced some CL, which differed depending on whether it was caused by IBG, WE, or DBG (Fig 4 and S1 Fig). As measured by the dECA index, CL was 54 ± 2.8% for IBG, 28 ± 2.4% for WE, and 4 ± 0.7% for DBG. CL-IBG was distributed evenly throughout the study area and, in 75% of the SPU, dECA was >30 (Fig 4a). CL-WE occurred more in some of the SPU; 75% of the SPU had a dECA < 40 (Fig 4b). CL-DBG was limited to a few SPU and, in 75% of the SPU, the dECA was < 4 (Fig 4c).

### Agropastoral system components in relation with connectivity loss in the dense grasslands

**Fig 5:**
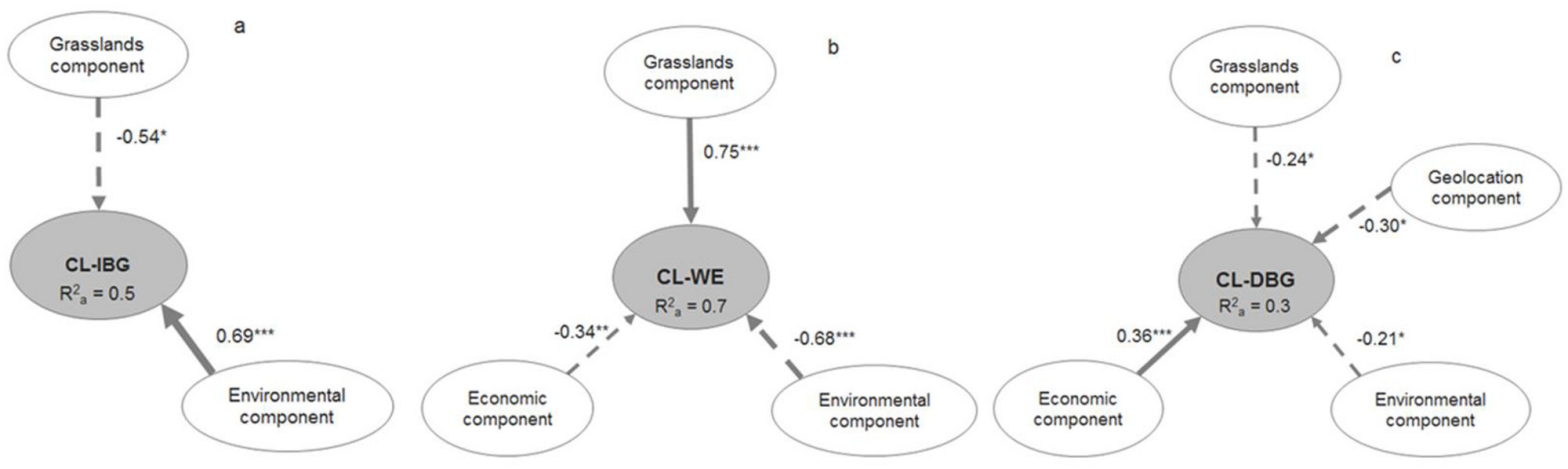
Final structural equation models for the relationships between the agropastoral system component and connectivity loss caused by a) increases in biomass and greenness (CL-IBG), b) woody encroachment (CL-WE), and c) decreases in biomass and greenness (CL-DBG). Arrow thickness reflects the contribution from the exogenous latent variables (agropastoral system components) to the endogenous latent variable (connectivity loss). The numbers on the lines denote the standardized regression coefficients (β), and the statistical significance is indicated as follows: * *p* ≤ 0.05, ** *p* ≤ 0.01 and *** *p* ≤ 0.001. R^2a^ represents the adjusted R^2^ of the endogenous variable.

The relationships between the components of the agropastoral system and connectivity loss differed depending on whether the losses were caused by IBG, WE, or DBG (Fig 5). The Goodness of Fit (GoF) index indicated that the overall fit of the models in explaining those relationships was high (GoF_CL-IBG_ = 0.8, GoF_CL-WE_ = 0.9, and GoF_CL-DBG_ = 0.7). Based on the adjusted R^2^, the proportion of the variance explained in each SEM model was 50% for CL-IBG (Fig 5a), 70% for CL-WE (Fig 5b), and 30% for CL-DBG (Fig 5c).

### Grassland component

A third of the 92 SPUs had >300 ha of dense grassland, another third had 100–300 ha, and one-third had <100 ha. The average dense grassland cover of the SPUs was 30 ± 2.1%. The current sheep and goat pressure was on average 0.9 ± 0.4 LLU * month * ha^-1^ during the grazing period, although most SPUs were not grazed by sheep or goats. Average current cattle pressure was 2.1 ± 0.3 LLU * month * ha^-1^. Since the 1930s, sheep and goat heads decreased by 79 ± 1.5% and cattle increased by 120 ± 11.3%.

**Fig 6:**
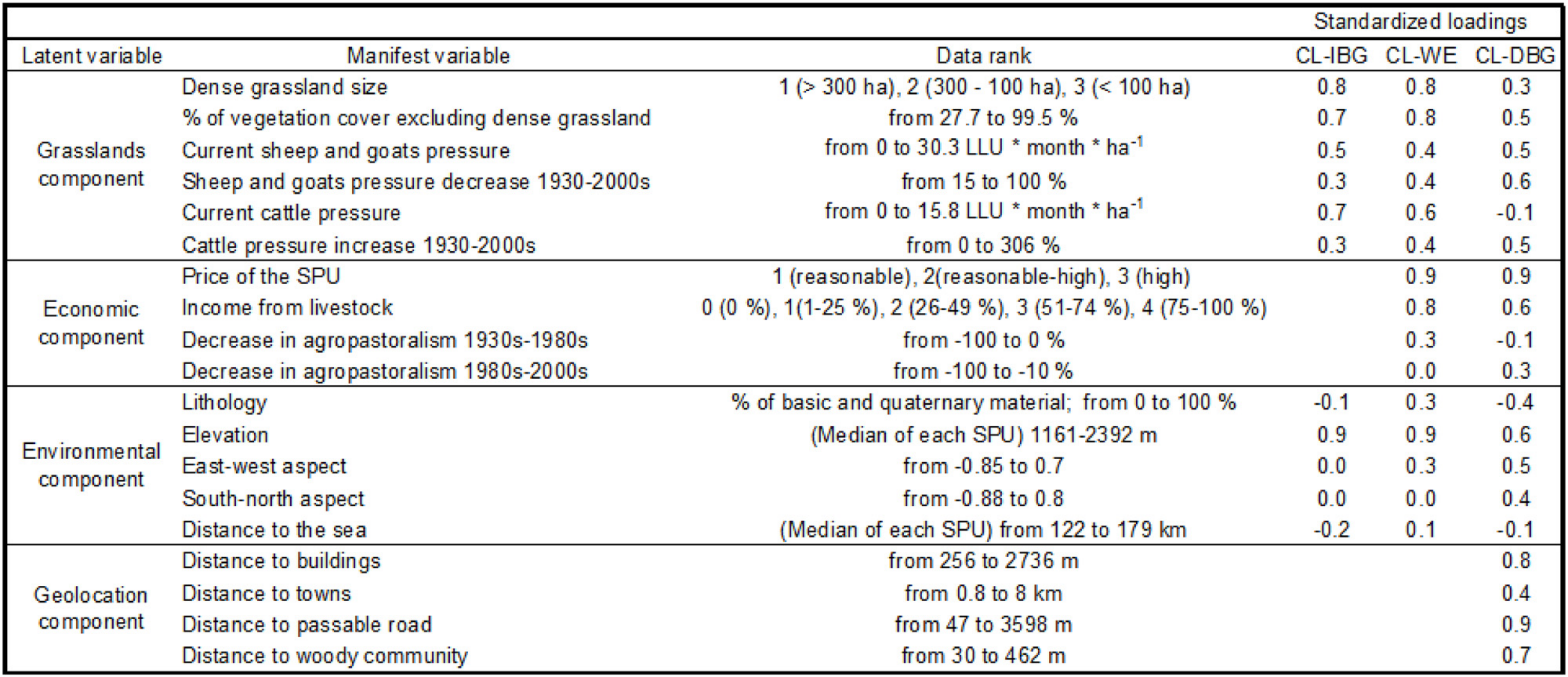
Standardized loadings (correlations) between each manifest variable and the corresponding exogenous latent variable. The correlations are for the connectivity losses caused by increases in biomass and greenness (CL-IBG), woody encroachment (CL-WE), and decreases in biomass and greenness (CL-DBG).

The grassland component and CL-IBG were negatively correlated (β = -0.54, *p* ≤ 0.05) (Fig 5a). The SPUs that had a high proportion and high coverage of dense grassland had the highest CL-IBG (Fig 6). SPUs that had high CL-IBG had low current cattle pressure, and low current sheep and goat pressure; however, between the 1930s and the 2000s, those SPUs experienced a small reduction in sheep and goats and a small increase in the number of cattle (Fig 6).

The grassland component and CL-WE were strongly and positively correlated (β = 0.75, *p* ≤ 0.001) (Fig 5b). SPUs that had a small coverage of dense grassland and a high proportion of vegetation that was not dense grassland had the highest CL-WE (Fig 6). SPUs that had high CL-WE had high current cattle and, to a lesser extent, sheep and goat pressure (Fig 6). Between the 1930s and the 2000s, the SPUs that experienced large reductions in sheep and goat pressure and large increases in cattle pressure had high CL-WE (Fig 6).

The grassland component and CL-DBG were weakly and negatively correlated (β = -0.24, *p* ≤ 0.05) (Fig 5c). The SPUs that had a high proportion and high coverage of dense grassland had the highest CL-DBG (Fig 6). CL-DBG increased in SPUs where sheep and goat pressure was low and cattle pressure was high (Tabla1). Between the 1930s and the 2000s, SPUs that experienced a small reduction in the numbers of sheep and goats and a small increase in the number of cattle had high CL-DBG (Fig 6).

### Social component

The average age of the stockbreeders in the 92 SPUs was 50 ± 0.9 years, and young stockbreeders had the highest levels of education. On average, 4 ± 0.4 stockbreeders shared an SPU, although some SPUs were shared by as many as 16 stockbreeders. Since the 1930s, on average, the human populations in the municipalities have decreased by 43 ± 4.8%. Almost all of the stockbreeders in the study area had a second job associated with tourism. Most of the tourism activities were associated with ski resorts in Alto Gállego County and with Ordesa and Monte Perdido National Park in Sobrarbe County. The social component defined by those manifest variables was not significantly correlated with CL caused by IBG, WE, or DBG.

### Economic component

Sixty percent of the stockbreeders who used the 92 SPUs felt that the prices that they paid for the use of the SPUs were reasonable. The average rank of the incomes from livestock breeding was 2.9 ± 0.1 (range = 0–4), which indicated that 50–74% of the stockbreeders’ salaries came from this source. The importance of agropastoral activities decreased by 48 ± 1.9% between the 1930s and the 1980s, and by 74 ± 2% between the 1980s and the 2000s. The economic component was negatively correlated with CL-WE (β = -0.34, *p* ≤ 0.01) and positively correlated with DBG (β = 0.36, *p* ≤ 0.001) (Fig 5b and 5c). The SPUs that had high rental fees and were used by stockbreeders with high incomes from livestock breeding had low CL-WE and high CL-DBG (Fig 6). SPUs in which agropastoralism had decreased most between the 1930s and the 1980s had high CL-WE; SPUs in which agropastoralism decreased the least between the 1980s and the 2000s had high CL-DBG.

### Environmental component

The average elevation of dense grasslands was 1881 ± 27 m a.s.l. Most grasslands in the 92 SPUs had a south-west aspect and 77% of the SPUs had >50% of its area on acidic material. On average, the SPUs were 147 ± 1.4 km from the Cantabric Sea, and distance to the sea was negatively correlated with annual rainfall. The environmental component was strongly and positively correlated with CL-IBG, and negatively correlated with CL-WE (β = 0.69, and β = -0.68 respectively; *p* ≤ 0.001) (Fig 5a and 5b). Elevation was the most important manifest variable that described the environmental component in the context of connectivity loss (Fig 6). High-elevation SPUs were the most important in the context of CL-IBG, and low elevation SPUs were most strongly associated with CL-WE. CL-IBG was high in the SPUs that were closest to the Cantabric Sea. Acidic lithology and a western aspect were positively correlated with CL-WE. The environmental component and CL-DBG were weakly and negatively correlated (β = -0.21, *p* ≤ 0.05) (Fig 5c), and the manifest variables that described the relationship were low elevation SPUs that had a high proportion of basic and quaternary materials, and SPUs in which western and northern aspects predominated (Fig 6).

### Geolocation component

On average, the 92 SPUs were 111 ± 7.1 m from a woody community, 3.5 ± 0.16 km from the nearest town, 954 ± 81 m from the nearest passable road, and 934 ± 52 m from the nearest building. The geolocation component and CL-DBG were weakly and negatively correlated (β = -0.3, *p* ≤ 0.05) (Fig 5c). The grasslands that had the highest CL-DBG were in SPUs that were near towns, passable roads, buildings, and woody communities (Fig 6).

## Discussion

In dense grasslands of the Central Pyrenees, Spain, we identified the components of the agropastoral system that were correlated with connectivity loss (CL) caused by increases in biomass and greenness (IBG), woody encroachment (WE), and decreases in biomass and greenness (DBG). The environmental and grassland components contributed to the three types of connectivity loss; particularly, land abandonment affected CL-WE and CL-IBG. The geolocation component explained a high proportion of the variation in where the intensification of land use and CL-DBG occurred. Although we expected that the economic component would have affected the three types of CL, the effect was only statistically significant for CL-WE and CL-DBG; CL-WE was associated with decreases in agropastoral activities, generally, and CL-DBG was associated with the concentration of land use. The social component had no direct effect on grassland connectivity loss, possibly, because this component acted at a broader scale; e.g., the regional level [67], and not at the grassland unit level [12, 68].

### Agropastoral system components correlated with CL-IBG

An increase in productivity indicated by IBG is not always associated with an improvement in vegetation quality [29]. IBG can be associated with negative effects such as increased grassland roughness and loss of nutritive quality for livestock [15, 28, 69]. The **environmental component** was strongly correlated with CL-IBG, which had occurred throughout the study area [15] and might indicate the succession of the vegetation toward climax communities. Climate warming can cause a rapid increase in the biomass and greenness of grasslands [70–73], and elements of the **grassland component**, such as grazing management, can aggravate this effect. CL-IBG was highest in SPUs with large extension and high proportion of dense grasslands, particularly, where current livestock pressure was low. Low livestock grazing can cause IBG [74, 75], and reduce species richness and forage quality [28, 76]. This effect was especially pronounced at high elevations and in remote areas, where most of the land abandonment has occurred, as the majority of livestock grazing, currently by cattle, is limited to low elevation and easily accessible areas [77, 78].

### Agropastoral system components correlated with CL-WE

Woody plant encroachment reduces the extension of grasslands and increases the fragmentation and isolation of them [5, 14, 20, 79], with negative consequences such as losses in grassland diversity and quality, and amount of livestock forage [80–82]. The CL in grasslands caused by WE was strongly correlated with the **environmental components**. The seriation of the vegetation toward a climax community, exacerbated by climate warming, can facilitate woody encroachment of grasslands [22, 73, 83]. CL-WE has occurred throughout the study area, especially at low elevations where grasslands are close to existing woody habitats [14, 68, 84, 85]. The absence of significant effects of the geolocation component might be related to measuring the distance to woody habitat as the average distance from the SPU to the nearest woody habitat, without consideration of the size or length of the perimeter of the woody community in the SPU, which can affect woody encroachment of neighboring grasslands. SPUs that had a westerly aspect and had been abandoned in recent decades, showed a high rate of woody encroachment [14, 86, 87].

The **grassland component** strongly affected CL-WE; small SPUs and those that had a high proportion of woody habitat were the most vulnerable to woody encroachment [14]. CL-WE occurred even in SPUs where cattle grazing pressure was high [23], possibly, because cattle preferred open and accessible grasslands and, consequently, shrub areas were less used [78, 88]. Thus, apparently, cattle pressure did not slow woody encroachment or CL caused by WE, at least in areas where cattle had enough forage outside shrub habitats. Grassland units that had a large reduction in sheep and goats since the 1930s, however, were most prone to high CL-WE, even though grazing by sheep did not fully counteract the expansion of mountain woody habitats [5, 89]. The **economic component** affected CL-WE; the most expensive SPUs had the fewest problems with woody encroachment, possibly, because these pastures were the most productive and the highest pressure of livestock actively grazing these areas would have slowed woody encroachment. In addition, CL-WE was associated with the reduction in agropastoralism between the 1930s and the 1980s and the increase in part-time farming, which led to the partial abandonment of the grasslands [34, 90]. Even so, the social component, especially the reduction in the human population since the 1930s, was not directly correlated with CL-WE at the SPU level.

### Agropastoral system components correlated with CL-DBG

The reduction in the extent and quality of livestock forage was a direct negative effect of DBG on the dense grasslands [7, 15]. The **economic component** had the greatest influence on CL-DBG; the more expensive grasslands and those associated with stockbreeders that had high incomes from livestock activities had the highest CL-DBG. In addition, CL-DBG was associated with the SPUs in which agropastoralism decreased the least between the 1980s and the 2000s, thus, the continuity of agropastoralism has not always had favorable consequences. The correlation between CL-DBG and the **grassland component** was associated with extensive grasslands that had low sheep and goat stocking rates and high cattle rates, where grazing has been centralized and overgrazing has damaged the grasslands [21, 91].

The **geolocation component** influenced CL-DBG; the most heavily grazed and degraded grasslands were in areas near passable roads and buildings. These areas were most accessible to stockbreeders to manage livestock, had salt licks and water for livestock, and refuges for shepherds and stockbreeders [85, 89, 92, 93]. The **environmental component** also had an effect, because some locations that had favorable characteristics were used most often by the livestock and, consequently, were the most degraded [94]. The most degraded areas were at low elevations and on basic and quaternary materials. Although the loss of grasslands associated with CL-DBG affected a relatively small area of the SPUs, the problem is significant because it affected the grasslands that had the highest quality and were the most accessible for livestock grazing [15].

### Recommendations for reducing the loss of dense grasslands

Most of the dense mountain grasslands in the Central Pyrenees were created by grazing livestock. Stockbreeders who manage the livestock and the grasslands are the most interested in preserving resilient grasslands [95]; however, their activities might have compromised the system [96]. Grazing type and pressure have a significant effect on the composition of vegetation [22, 28]; therefore, we believe that adequate management of grasslands and livestock based on the following suggestions should slow the loss of resilient grasslands, connectivity loss, and the increase in the vulnerability of the grasslands:

- Vulnerability of the grasslands caused by CL-IBG will persist if the grasslands proceed toward climax vegetation because of land abandonment and climate warming. Livestock grazing might slow the process if grazing is distributed more evenly throughout the grasslands, even if they are distant from accessible areas and difficult to reach or manage [97]. For that reason, we recommend an increase in sheep and goat flocks led by shepherds, because they are the most effective at grazing the most inaccessible pastures.
- Particularly, in grasslands that are close to woody habitats, WE might lead to climax vegetation because of land abandonment and climate warming. Government policies against the removal of shrubs through prescribed fires and mechanized clearing might have contributed to the increase in woody encroachment; therefore, these prohibitions should be reviewed. Although grazing alone cannot prevent shrub encroachment in grasslands [36, 89, 98], sheep and goats led by shepherds can slow the process [22, 99].
- The degradation of the grasslands caused by DBG, and the resulting connectivity loss, occurred in overgrazed or overused areas of the SPU. Proper management of the lands and livestock; e.g., shepherds leading and caring for their livestock *in situ*, and the appropriate placement of salt licks and water sources, can prevent overuse and degradation.

In addition, financial incentives can influence stockbreeders´ decisions [95] and management of the grasslands [100], although that was not demonstrated in our analyses. The most significant changes in livestock farming practices came before European Common Agricultural Policy subsidies [15], apparently, because the cost of labor for managing sheep is higher than it is for cattle. The increase in cattle farming and the decrease in agropastoral activity since the 1930s, have led to cattle being concentrated in areas where managing the animals is easiest, which has contributed to grassland degradation [14, 78]. Cattle do not graze the most remote and inaccessible areas as do sheep led by a shepherd [78]. An increase in the number of grazers is not as important as is an increase in grazing pressure by different types of livestock (cattle, sheep, goats, and mares), which can graze different types of grasslands and contribute to an increase in biodiversity [3, 17, 22, 78, 101]. An increase in the number of sheep and goat herders is desirable, but the shortage of labor caused by rural depopulation has resulted in a substantial reduction in this profession. The recent renewal in rural areas and increases in job demand stemming from the recent economic crisis has not reversed the trend, probably, because the new population works in tourism related jobs [102] and the limited social bonds between native residents and newcomers breakdown the socioeconomic structure of the mountain rural societies.

Resilient grasslands have been lost to the point where other types of vegetation have appeared. Once a threshold has been exceeded, it is very difficult to bring the grasslands back [25, 78, 103], either because it is too costly or it is forbidden because of habitat protection laws [104]. The increases in the vulnerability of the grasslands caused by CL-IBG, CL-WE, and CL-DBG have specific relationships with the components of the agropastoral system; therefore, all components and the causes of grassland vulnerability must be considered in management plans. Top-down management systems cannot always resolve resource-use conflicts [105]; however, bottom-up management systems alone will not work [96]. Therefore, an integrated management plan that involves the participation of stakeholders at all levels will be optimal [22, 95].

## Conclusions

When studying agropastoral systems in the context of grassland management, we have to consider the various changes that are occurring in grassland physiognomy and physiology, including CL-IBG, CL-WE, and CL-DBG. Those changes have different origins and, in each case, different agropastoral system components are in effect; e.g., environmental, grassland, economic, and geolocation components are acting at the SPU level. A proper integrated management plan for grasslands requires that all of the components are taken into account. In our study, the changes that were related to the seriation of the vegetation (CL-WE and CL-IBG) were strongly correlated with the environmental component. The grassland component might be the key in accelerating or slowing CL-WE and CL-IBG, especially in terms of livestock pressure. CL-DBG was affected by the economic component and the geolocation of mountain summer pastures, which indicated direct anthropogenic activity has influenced this connectivity loss.

## Supporting Information

S1 Fig Spatial distribution of the connectivity loss in the grasslands of the agropastoral systems in the Central Pyrenees, Spain. White lines in each map represent the boundaries of the summer pasture units (SPU): a) dense grassland size (ordinal values in brackets are the values used in the SEM models) and the proportion (%) of the vegetation cover excluding dense grasslands. Connectivity loss (expressed as the difference in the ECA index between the 1980s and the 2000s) in dense grasslands caused by b) increases in biomass and greenness (IBG), c) woody encroachment (WE), and d) decreases in biomass and greenness (DBG).
(TIF)

S2 Fig Spatial distribution of the grassland component of the agropastoral systems in the Central Pyrenees, Spain. White lines in each map represent the boundaries of the summer pasture units (SPU): current livestock pressure (a, c) and change (%) in the number of livestock head between the 1930s and the 2000s (b, d) for sheep and goats (a, b) and cattle (c, d). The values of a) and c) are presented as large livestock units (LLU) per month and hectare (ha).
(TIF)

S3 Fig Spatial distribution of the social component of the agropastoral systems in the Central Pyrenees, Spain. White lines in each map represent the boundaries of the summer pasture units (SPU) (values used in the SEM models are indicated in brackets).
(TIF)

S4 Fig Spatial distribution of the economic component of the agropastoral systems in the Central Pyrenees, Spain. White lines in each map represent the boundaries of the summer pasture units (SPU) (values used in the SEM models are indicated in brackets).
(TIF)

S5 Fig Spatial distribution of the geolocation component of the agropastoral systems in the Central Pyrenees, Spain. White lines in each map represent the boundaries of the summer pasture units (SPU). Average distance from the SPU to buildings, towns, roads, and woody plant communities.
(TIF)

S6 Fig Spatial distribution of the environmental component of the agropastoral systems in the Central Pyrenees, Spain. White lines in each map represent the boundaries of the summer pasture units (SPU) (values used in the SEM models are indicated in brackets).
(TIF)
