# Supplementary figures and images for: Influence of Agropastoral System Components on Mountain Grassland Vulnerability Estimated by Connectivity Loss
Source: PLoS One. 2016 May 12;11(5):e0155193. doi: 10.1371/journal.pone.0155193 (PMC4865193; doi:10.1371/journal.pone.0155193)

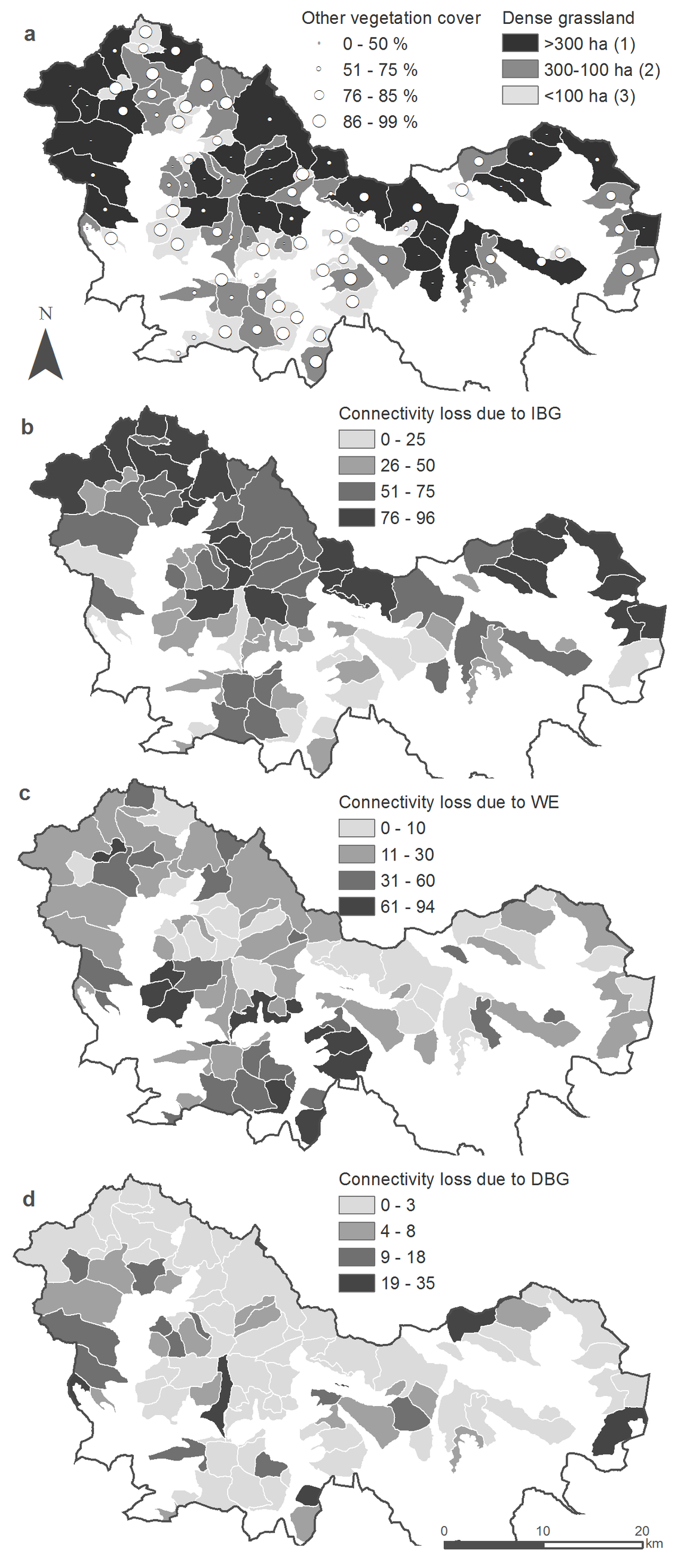

Supplement: S1 Fig — White lines in each map represent the boundaries of the summer pasture units (SPU): a) dense grassland size (ordinal values in brackets are the values used in the SEM models) and the proportion (%) of the vegetation cover excluding dense grasslands. Connectivity loss (expressed as the difference in the ECA index between the 1980s and the 2000s) in dense grasslands caused by b) increases in biomass and greenness (IBG), c) woody encroachment (WE), and d) decreases in biomass and greenness (DBG). (TIF) [file pone.0155193.s001.tif]

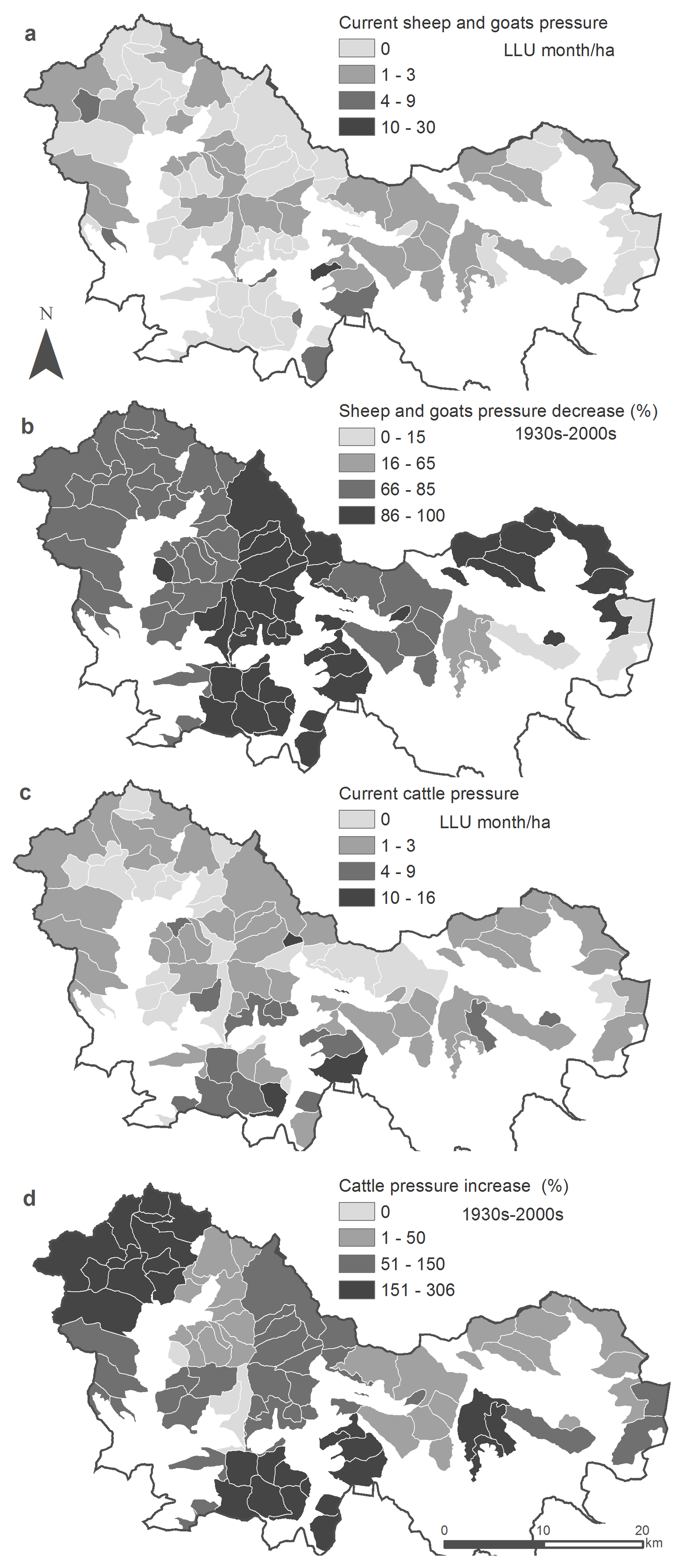

Supplement: S2 Fig — White lines in each map represent the boundaries of the summer pasture units (SPU): current livestock pressure (a, c) and change (%) in the number of livestock head between the 1930s and the 2000s (b, d) for sheep and goats (a, b) and cattle (c, d). The values of a) and c) are presented as large livestock units (LLU) per month and hectare (ha). (TIF) [file pone.0155193.s002.tif]

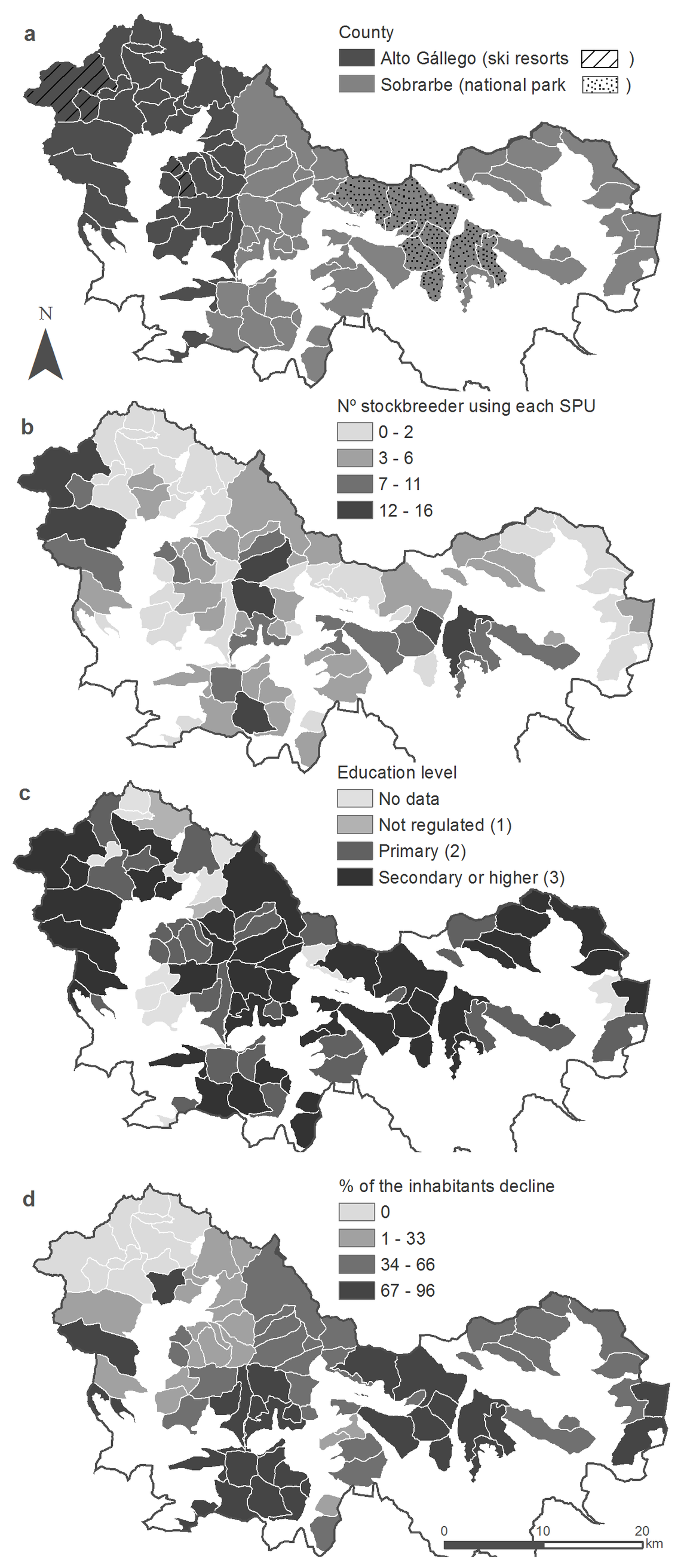

Supplement: S3 Fig — White lines in each map represent the boundaries of the summer pasture units (SPU) (values used in the SEM models are indicated in brackets). (TIF) [file pone.0155193.s003.tif]

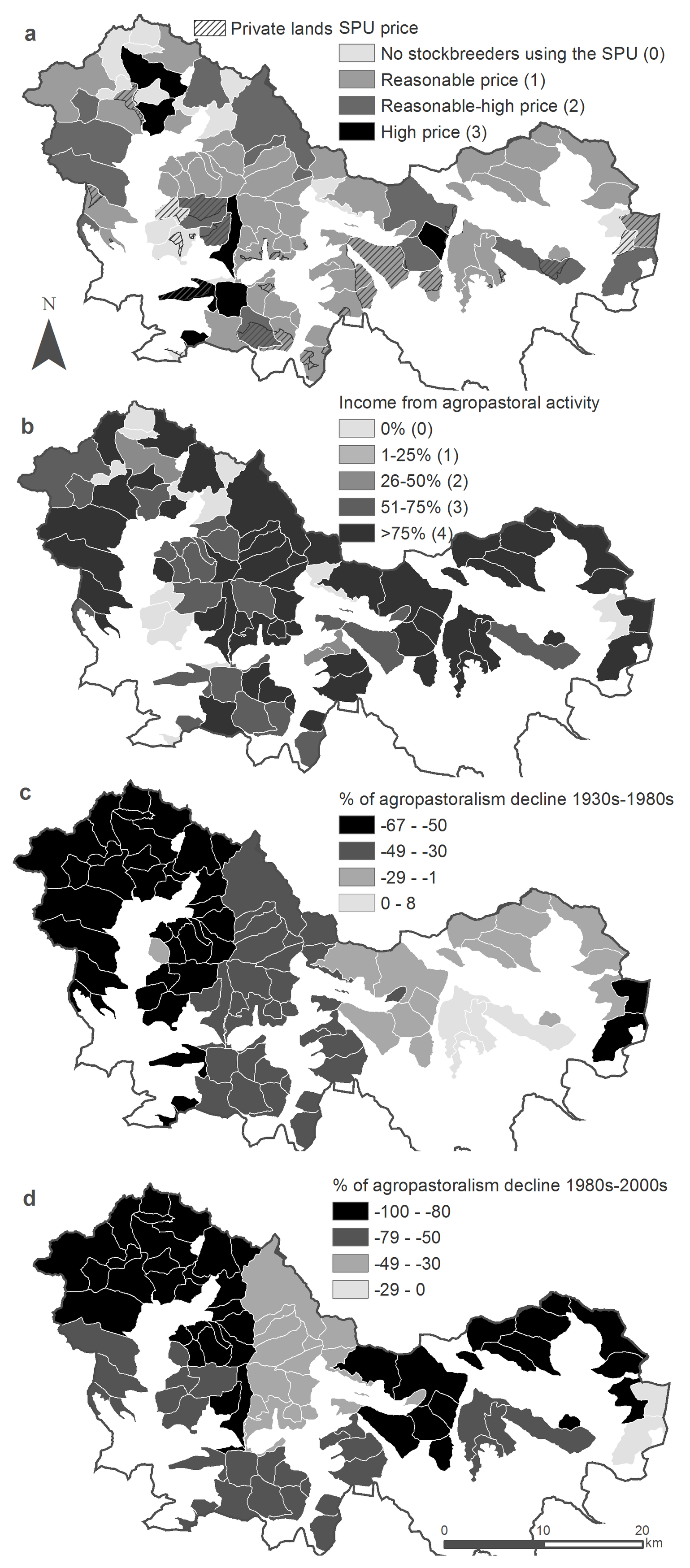

Supplement: S4 Fig — White lines in each map represent the boundaries of the summer pasture units (SPU) (values used in the SEM models are indicated in brackets). (TIF) [file pone.0155193.s004.tif]

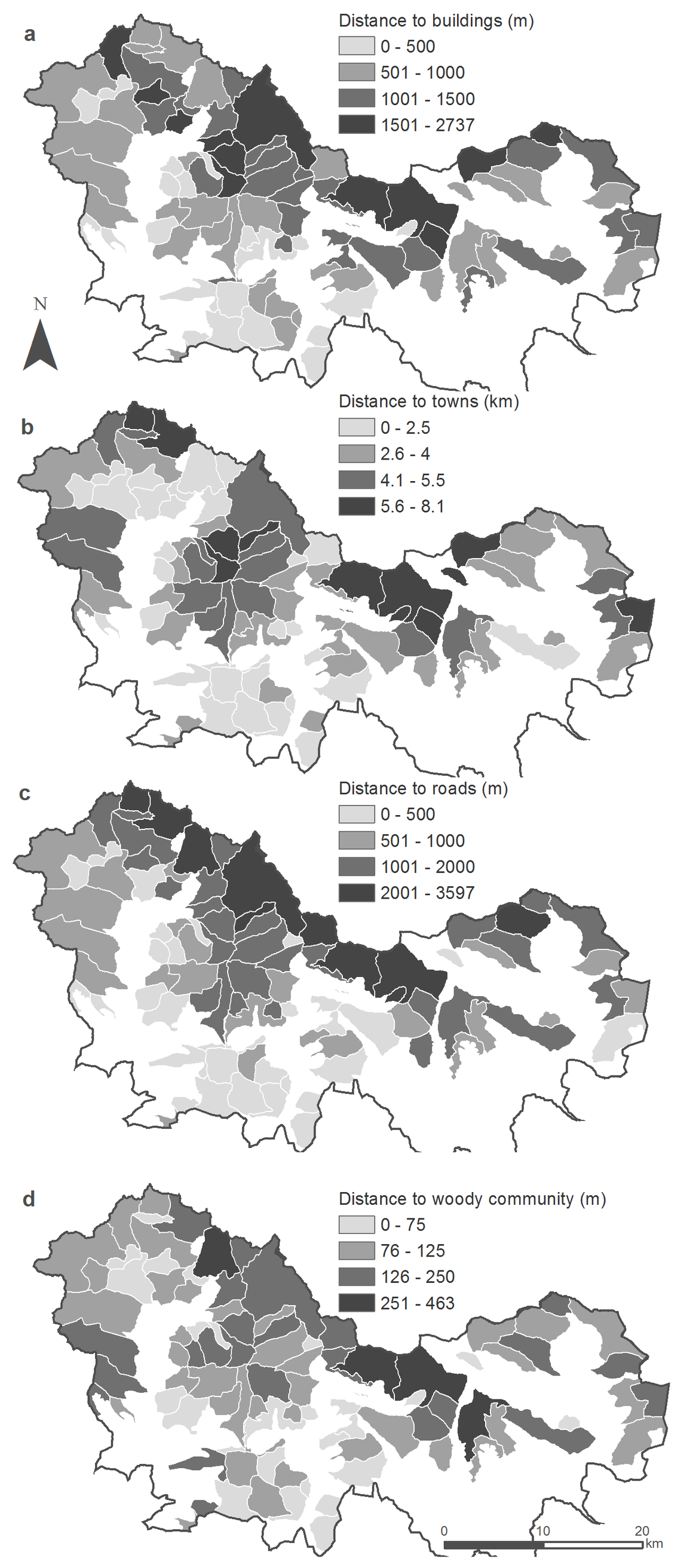

Supplement: S5 Fig — White lines in each map represent the boundaries of the summer pasture units (SPU). Average distance from the SPU to buildings, towns, roads, and woody plant communities. (TIF) [file pone.0155193.s005.tif]

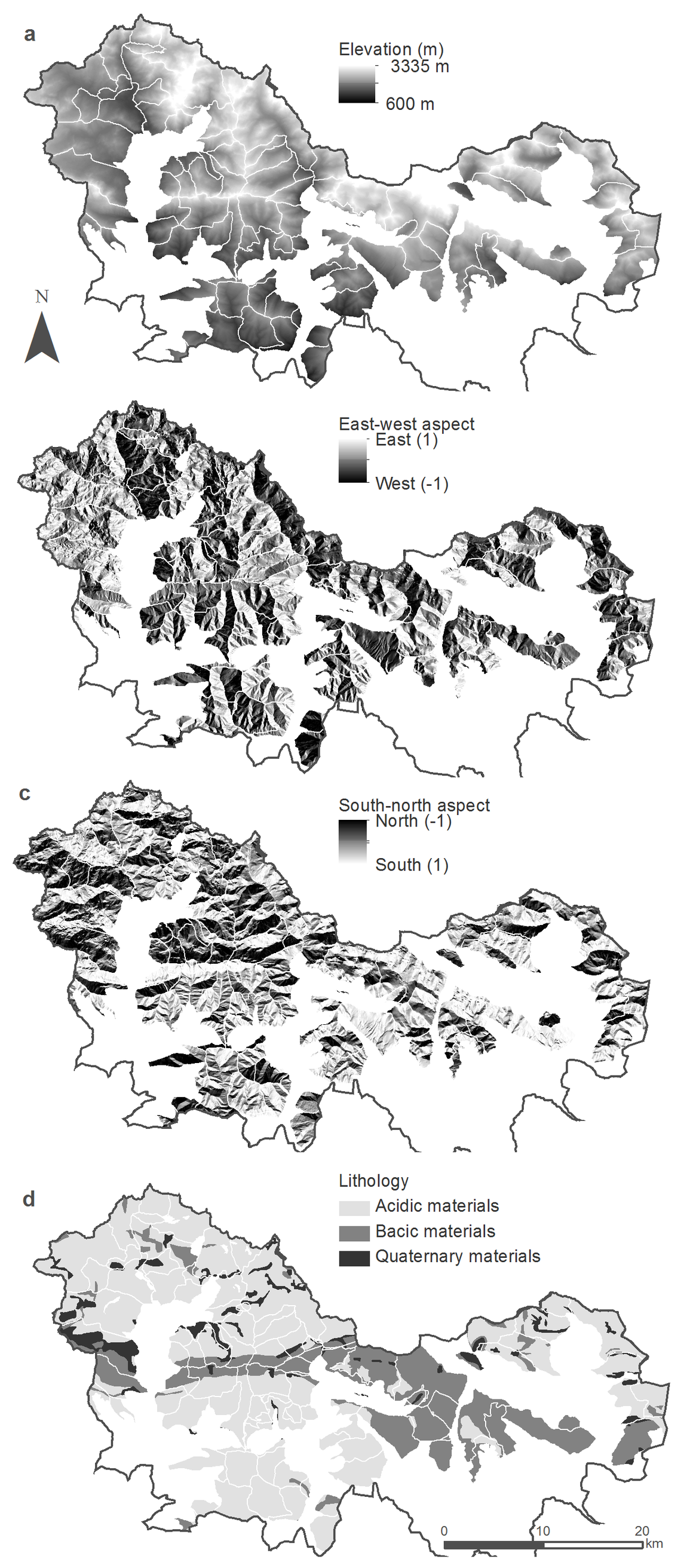

Supplement: S6 Fig — White lines in each map represent the boundaries of the summer pasture units (SPU) (values used in the SEM models are indicated in brackets). (TIF) [file pone.0155193.s006.tif]
